# Relationships between the body size attitudes of fathers and mothers and those of their four-year-old sons and daughters

**DOI:** 10.1186/2050-2974-2-S1-O33

**Published:** 2014-11-24

**Authors:** Stephanie R Damiano, Susan J Paxton, Karen J Gregg, Emma C Spiel, Siân A McLean

**Affiliations:** 1 School of Psychological Science, La Trobe University, Melbourne, Australia

## 

Despite evidence that parents can influence the development of body dissatisfaction and disordered eating in older children and adolescents, few studies have examined relationships between parents' body size attitudes and related behaviours and the body size attitudes of pre-school children. In particular, there has been little focus on relationships between fathers' attitudes and the attitudes of their young sons, the central aim of this study. Participants were 279 four-year-old children (46% boys) and their mothers (N= 270) and fathers (N= 205). Children were interviewed to assess their body size attitides and body ideals. Parents completed questionnaires assessing their weight-based attitudes, internalisation of media and athletic body ideals, body image and dieting. Paternal concern about their son's weight was associated with boys' body dissatisfaction (wanting to be larger). For boys, both negative and positive body size stereotyping were associated with paternal negative attitudes towards obese people. For girls, positive body size stereotyping was associated with maternal dieting. Results suggest that even at young ages, children may be influenced by parental attitudes. Fathers may be conveying the muscular ideal to their sons and mothers may be conveying the thin ideal to their daughters.

This abstract was presented in the **Parental Roles in Prevention and Support** stream of the 2014 ANZAED Conference.

